# Parvovirus B19 infection presenting with severe erythroid aplastic crisis during pregnancy in a woman with autoimmune hemolytic anemia and alpha-thalassemia trait: a case report

**DOI:** 10.1186/s13256-015-0542-7

**Published:** 2015-03-12

**Authors:** Chi-Ching Chen, Chin-Shan Chen, Wei-Yao Wang, Jui-Shan Ma, Hwei-Fan Shu, Frank S Fan

**Affiliations:** 1 Department of Internal Medicine, Feng Yuan Hospital, No. 100, Ankang Road, Fengyuan District Taichung City, 420 Taiwan; 2 Department of Gynecology and Obstetrics, Feng Yuan Hospital, No. 100, Ankang Road, Fengyuan District Taichung City, 420 Taiwan; 3 Section of Infectious Disease, Department of Internal Medicine, Feng Yuan Hospital, No. 100, Ankang Road, Fengyuan District Taichung City, 420 Taiwan; 4 Department of Pediatrics, Feng Yuan Hospital, No. 100, Ankang Road, Fengyuan District Taichung City, 420 Taiwan; 5 Department of Anatomical Pathology, Feng Yuan Hospital, No. 100, Ankang Road, Fengyuan District Taichung City, 420 Taiwan; 6 Division of Hematology/Oncology, Department of Internal Medicine, Feng Yuan Hospital, No. 100, Ankang Road, Fengyuan District Taichung City, 420 Taiwan

**Keywords:** Parvovirus B19, Pregnancy, Autoimmune hemolytic anemia, Transient aplastic crisis, Intravenous immunoglobulin

## Abstract

**Introduction:**

Parvovirus B19 virus commonly causes subclinical infection, but it can prove fatal to the fetus during pregnancy and cause severe anemia in an adult with hemolytic diseases. We present the case of a woman with autoimmune hemolytic anemia who was diagnosed with parvovirus B19-induced transient aplastic crisis during her second trimester of pregnancy and faced the high risk of both fetal and maternal complications related to this specific viral infection. To the best of our knowledge, the experience of successful intravenous immunoglobulin treatment for B19 virus infection during pregnancy, as in our case, is limited.

**Case presentation:**

A 28-year-old and 20-week pregnant Chinese woman with genetically confirmed alpha-thalassemia trait was diagnosed with cold antibody autoimmune hemolytic anemia and suffered from transient aplastic crisis caused by B19 virus infection. She received intravenous immunoglobulin treatment to reduce the risk of hydrops fetalis. Her peripheral blood reticulocyte percentage recovered, but anemia persisted, so she underwent several courses of high dose intravenous dexamethasone for controlling her underlying hemolytic problem. Finally, her hemoglobin levels remained stable with no need of erythrocyte transfusion, and a healthy baby boy was naturally delivered.

**Conclusions:**

Parvovirus B19 virus infection should be considered when a sudden exacerbation of anemia occurs in a patient with hemolytic disease, and the possible fetal complications caused by maternal B19 virus infection during pregnancy should not be ignored. Close monitoring and adequate management can keep both mother and fetus safe.

## Introduction

Most parvovirus B19 virus (B19 virus for short in this report) infection causes asymptomatic or only mild illness, such as erythema infectiosum, polyarthropathy syndrome or transient reticulocytopenia in healthy adults or children, but can sometimes be responsible for life-threatening diseases. The cytopathic effect of B19 virus in patients with rapid erythrocytes production results in profuse extinction of proerythroblasts. Failure of differentiation from proerythroblast into later stage erythroid precursors leads to transient aplastic crisis (TAC) [1]. When B19 virus infection occurs during pregnancy, transplacental transmission of B19 virus to the fetus can induce hydrops fetalis or fetal loss due to severe anemia and cardiac failure [2]. Here we report a rare case of severe anemic crisis due to B19 virus infection in a 20-week pregnant woman with alpha-thalassemia trait and possible pregnancy-related cold agglutinin hemolysis.

## Case presentation

This 28-year-old and 20-week pregnant Chinese woman with genetically confirmed alpha-thalassemia trait and a current obstetric record of gravida 3, para 0, artificial abortion 1 and ectopic pregnancy 1 (G3P0AA1E1 by Gravida/para/abortus (GPA) system) presented to our emergency unit (EU) due to headache, chills, fever and general soreness for one day. At our EU, her physical examination revealed a fever up to 38.5°C, mild tachypnea with a respiratory rate up to 21 breaths/min and bilateral lower back knocking pain. Her obstetric ultrasound revealed 20 weeks gestational age, normal placenta location and a fetal heart beat between 140 and 150/min. No vaginal bleeding was noted.

**Figure 1:**
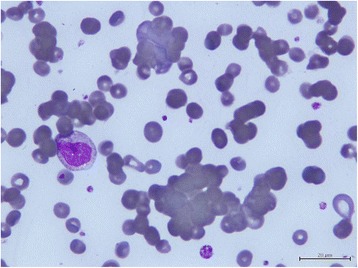
**Peripheral blood smear.** Aggregation of erythrocytes was noted in the peripheral blood smear just before dexamethasone treatment.

Her laboratory data showed pyuria (urine white blood cell count 30 to 50/high power field) and severe peripheral blood pancytopenia: white blood cell (WBC) count 2200/mm^3^ with an absolute neutrophil count (ANC) of 1780/mm^3^, hemoglobin 5.5g/dL, mean corpuscular volume (MCV) 119.4fl and platelet count 116,000/mm^3^. Aggregation of erythrocytes (Figure 1) and low reticulocyte percentage (0.1%) were detected in peripheral blood smear. Both direct and indirect antiglobulin tests were strongly positive for antibodies against erythrocytes. The autoantibody was found to be of the cold type. A subsequent laboratory investigation revealed 1:32(+) of cold hemagglutinin titer, raised lactate dehydrogenase (314U/L) and low levels of complement 3 (55.5mg/dL) and complement 4 (10mg/dL).

**Figure 2:**
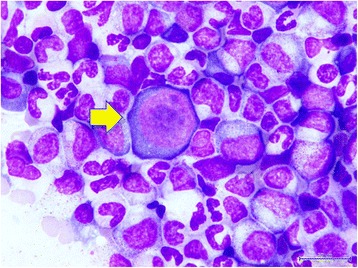
**Cytology of bone marrow.** The yellow arrow indicates a giant pronormoblast in the bone marrow.

**Figure 3:**
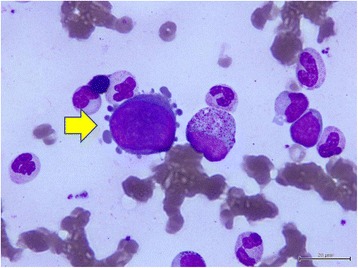
**Cytology of bone marrow.** The yellow arrow indicates a giant pronormoblast with ‘dog ear’ cytoplasmic projections in the bone marrow.

**Figure 4:**
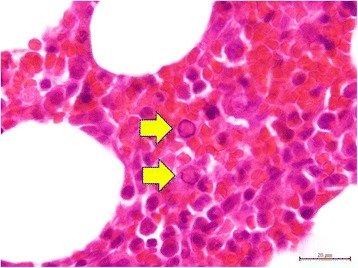
**Bone marrow core biopsy.** Parvovirus B19 intranuclear viral inclusions (clear area) leading to chromatin marginalization to the vicinity of the nuclear membrane within two pronormoblasts indicated by the yellow arrows.

**Figure 5:**
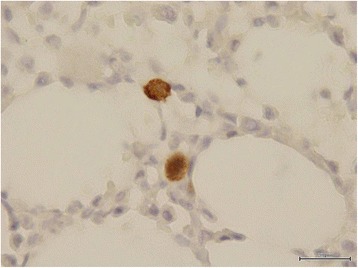
**Immunohistochemical staining of bone marrow core biopsy.** Immunohistochemical staining for parvovirus B19 using monoclonal antibody NCL-PARVO (Novocastra^TM^, Leica Microsystems, Newcastle upon Tyne, United Kingdom) specific for viral antigens VP1 and VP2 in bone marrow core biopsy specimen.

Her initial bone marrow cytology showed myeloid hyperplasia and only very few erythroid precursors with erythroblasts in abnormal megaloblastic change (Figure 2), some of which presented with pseudopods or ‘dog ears’ (Figure 3), and almost no erythroid maturation beyond basophilic normoblasts. Her bone marrow biopsy revealed scattered erythroblasts displaying homogeneous ground glass intranuclear viral inclusions (Figure 4) and positive nuclear immunostaining of B19 virus (Figure 5). Polymerase chain reaction for B19 virus DNA was positive in specimens from her bone marrow, plasma and nasal cavity. Antibodies against Epstein-Barr virus (EBV) were not checked because she did not present with the symptoms and signs of infectious mononucleosis or lymphoproliferative disorders present in most reported cases of EBV-associated hemolytic anemia.

**Figure 6:**
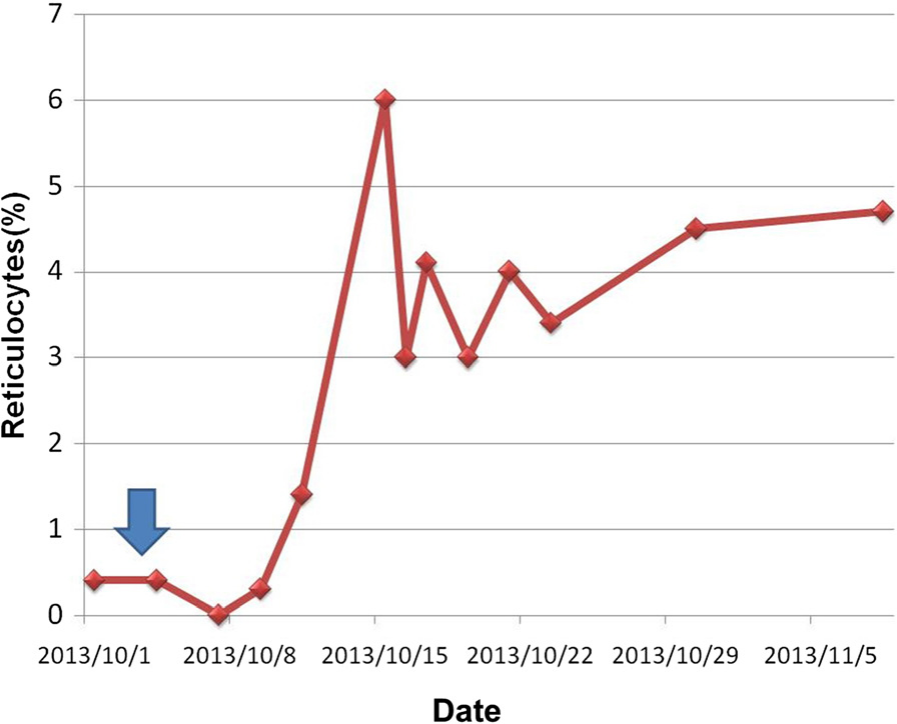
**Rapid response of intravenous human immunoglobulin treatment.** Effects of intravenous human immunoglobulin (IVIG) on reticulocytes percentage in peripheral blood during treatment course. The blue arrow stands for day one of IVIG 0.4gm/kg/day for five constitutive days.

**Figure 7:**
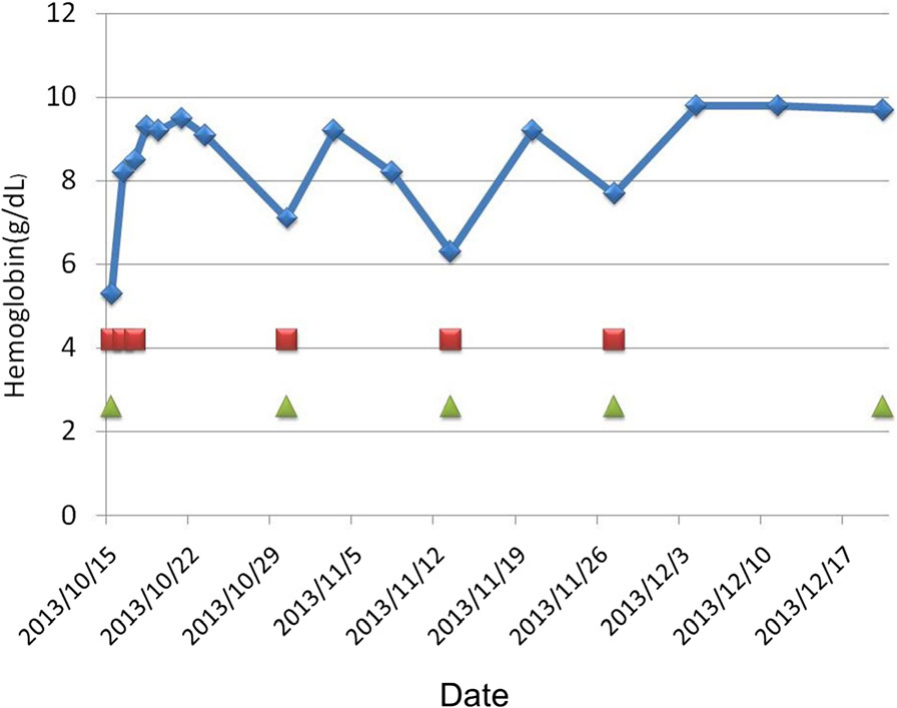
**Hemoglobin correction and dexamethasone treatment.** Effects of pulse high dose dexamethasone treatment on hemoglobin level. The green triangles stand for day one of dexamethasone 40mg/day for four constitutive days. The red squares stand for erythrocytes transfusion.

TAC caused by B19 virus infection in a pregnant woman with cold antibody autoimmune hemolytic anemia (AIHA) was diagnosed. She received intravenous immunoglobulin (IVIG) injection (CSL Limited, Parkville, Australia), 0.4gm/Kg/day for five days, for eradication of viremia and prophylaxis of occurrence of hydrops fetalis. Although her peripheral blood reticulocyte percentage increased dramatically after IVIG treatment (Figure 6), her hemolytic anemia did not improve much. In order to control her autoimmune hemolysis, intravenous high dose dexamethasone (Astar Chem. & Pharm., Hsinchu, Taiwan), 40mg/day for four days, was prescribed. This treatment was repeated every two weeks for four cycles and then shifted to a monthly oral schedule. She was discharged in a stable condition after the fourth intravenous treatment and received careful follow-up study in our outpatient clinic. No more erythrocytes were transfused after the fifth course of high dose dexamethasone (Figure 7) in spite of persistent mild anemia. During her hospitalization and subsequent outpatient clinic visits, obstetric ultrasound did not reveal any evidence of fetal anemia and hydrops fetalis. Four months later, a normal looking healthy baby boy (Apgar score 8 at one minute and 9 at five minutes, body weight 3240gm) was naturally delivered with vacuum extraction aid. After delivery of the baby, her AIHA quickly resolved.

## Discussion

Whether our patient’s B19 virus in her second trimester pregnancy was a new infection or a reactivation of a persisting previous infection cannot be surely answered, since the persistence of infection in the bone marrow has been reported in immunocompetent individuals several years after primary infection [3]. B19 virus infection causes a five to 10-day cessation of erythrocytic production, but the life span of normal erythrocytes is about 120 days, so B19 virus infection usually is asymptomatic or mildly symptomatic with self-limited anemia in people without hemolytic diseases [4]. In patients with expanding erythrocyte production caused by the shortened life span of erythrocytes due to an underlying hemolytic problem, such as spherocytosis, sickle cell anemia, autoimmune hemolytic anemia, thalassemia and glucose-6-phosphate dehydrogenase deficiency, B19 virus infection could induce TAC with severe anemia [2,5]. Thrombocytopenia and neutropenia have also been reported in patients during or after B19 virus infection [2,4,6].

Most patients with TAC had a good prognosis after erythrocyte transfusion and adequate monitoring until their bone marrow recovered, while IVIG is recommended for patients at risk of severe complications or with immunodeficiency [2,4,5]. Rapid efficacious reticulocyte recovery and hemoglobin correction after only one IVIG course has also been reported with only a few side effects [7]. Maternally administrated IVIG therapy during pregnancy for improving maternal and fetal outcome in severe B19 virus-induced disorders has also been reported in two cases without obvious complications [8,9]. Weekly fetal ultrasound examination including estimation of middle cerebral artery peak systolic velocity (MCA-PSV) for timely detection of possible fetal anemia and hydrops fetalis during a post-exposure period of 12 to 20 weeks was highly recommended [10].

In our patient, a 20-week pregnant woman with alpha-thalassemia trait and autoimmune hemolytic anemia, B19 virus infection not only caused TAC in the mother, but could also have led to severe anemia, hydrops and even death of the fetus, as cautioned by experts when maternal infection occurred between 17 and 24 weeks of gestation [11]. Therefore we arranged immediate IVIG treatment in addition to erythrocyte transfusion. Our patient responded well to IVIG treatment since her reticulocyte dramatically recovered. However, her anemia due to cold agglutinin disease persisted. So far, only corticosteroids and rituximab (both in pregnancy risk class C) were considered eligible for treating cold agglutinin disease during the third trimester of pregnancy.

Although corticosteroid treatment in the first trimester is well-known to increase the risk of fetal orofacial clefts, and the safety of mother and fetus receiving corticosteroids for treating idiopathic thrombocytopenic purpura in the third trimester of pregnancy, even with high dose intravenous methylprednisolone, was previously confirmed [12], the experience of corticosteroid pulse therapy in the second trimester was limited to the best of our knowledge. However, long term inhibition of neonatal B-lymphocyte development caused by maternal administration of rituximab in the third trimester of pregnancy has been reported [13]. Even though the immune impairment was reversible and no infection-related complications took place according to the literature, our patient refused rituximab injection for fear of damage to the baby. In spite of the generally poor effect of corticosteroid therapy in treating cold antibody AIHA [14], our high dose dexamethasone policy starting in our patient’s second trimester of pregnancy controlled her cold agglutinin disease satisfactorily, without notable side effects, in both the mother and her baby.

## Conclusions

B19 virus infection in a pregnant woman with increased erythropoietic demand from alpha-thalassemia trait and cold AIHA resulted in a TAC in the mother and put the fetus at risk of development of hydrops fetalis. Prompt IVIG injection eradicated the virus rapidly, resolved TAC in the mother and prevented further dangerous fetal infection. Supportive erythrocytes transfusion and intensive high dose dexamethasone treatment maintained hemoglobin at stable levels. Safe delivery of a healthy baby was happily achieved.

### Patient perspective

Both my husband and I felt satisfactorily about the rapid recovery of my hematopoietic function and the safe delivery of a healthy baby boy. We would like to express our great appreciation to our doctors for their efficient and correct diagnosis and prompt management. Although the rather expensive intravenous immunoglobulin was not reimbursed for transient aplastic crisis according to the general health insurance policy in our country and we had to pay for it by ourselves, we thought it was worthwhile since nothing was more important than our health.

## Consent

Written informed consent was obtained from the patient for publication of this case report and accompanying images. A copy of the written consent is available for review by the Editor-in-Chief of this journal.

## Abbreviations

AIHA: Autoimmune hemolytic anemia
ANC: Absolute neutrophil count
EBV: Epstein-Barr virus
EU: Emergency unit
GAP: Gravida/para/abortus
IVIG: Intravenous immunoglobulin
MCA-PSV: Middle cerebral artery peak systolic velocity
MCV: Mean corpuscular volume
TAC: Transient aplastic crisis
WBC: White blood cell
